# Health Resort Treatment Improves Functioning and Physical Performance in Long COVID Patients: A Retrospective Study

**DOI:** 10.3390/healthcare12232344

**Published:** 2024-11-23

**Authors:** Grzegorz Onik, Katarzyna Knapik, Magdalena Dąbrowska-Galas, Karolina Sieroń

**Affiliations:** 1 Department of Physical Medicine, School of Health Sciences in Katowice, Medical University of Silesia in Katowice, 40-055 Katowice, Poland; kknapik@sum.edu.pl (K.K.); ksieron@sum.edu.pl (K.S.); 2 Department of Kinesitherapy and Special Methods, School of Health Sciences in Katowice, Medical University of Silesia in Katowice, 40-055 Katowice, Poland; mdabrowska-galas@sum.edu.pl

**Keywords:** long COVID, fatigue, physical performance, functional status, health resort treatment, balneology, rehabilitation

## Abstract

Background/Objectives: The physical performance and functional status of individuals with long COVID may be altered. Health resort treatment comprises balneology, exercises, physical medicine modalities, and climate therapy. Complex treatment in a sanatorium may have a positive effect on long COVID patients. This study assessed functional status, physical performance, and fatigue in people with long COVID that qualified for the health resort treatment and its efficacy in this group of patients. Methods: A retrospective review of the medical records of 116 patients (66 women and 50 men) undergoing health resort treatment for long COVID in 2021 at the Rehabilitation Hospital and Sanatorium “Gwarek” in Goczałkowice-Zdrój (Poland) was conducted. Data were collected between March and May 2024. Their functional status, physical performance, and level of fatigue were assessed twice: before and after the treatment. Results: After the health resort treatment, their physical performance (10.41 points ± 1.84 points vs. 11.57 points ± 0.94 points; *p* < 0.00001) and functional status (2.13 points ± 0.88 points vs. 1.23 points ± 0.62 points; *p* < 0.00001) improved. Their fatigue (4.83 points ± 2.38 points vs. 2.15 points ± 1.31 points; *p* < 0.00001) level was diminished after the treatment. Conclusions: Fatigue was of moderate intensity in the long COVID patients that qualified for the health resort treatment. Most of the long COVID patients reported mild functional limitations, whereas their physical performance was undisturbed. Health resort treatment improved functioning in patients with persistent COVID-19 symptoms by reducing fatigue, improving their functional capacity and physical performance. It should be recommended as a supplement to the standard treatment because of its complexity.

## 1. Introduction

In COVID-19 survivors, various persistent symptoms of SARS-CoV-2 infection may be observed [1]. According to the World Health Organization, symptoms usually occur three months from the onset of COVID-19 and last for at least two months. Moreover, another diagnosis cannot explain their presence [2]. Fatigue and dyspnea are the most commonly listed long COVID symptoms. Fatigue is a distressing, persistent feeling of weariness, tiredness, or exhaustion that is not alleviated by rest and is not proportional to recent activity levels. It diminishes individuals’ activities, impairs their cognitive functions, and reduces their quality of life [3,4,5,6,7,8]. The pathomechanism of long COVID is still debatable. Few hypotheses have explained the persistent COVID-19 symptoms in convalescents [9,10]. Nevertheless, the underlying mechanism of fatigue in COVID-19 survivors is assumed to be central nervous system disturbances [2] resulting from the insult of the brain’s glymphatic system, leading to cerebrospinal fluid congestion [4]. Moreover, it is also postulated that autonomic dysfunction determines fatigue in COVID-19 convalescents [11]. To date, different strategies have been implemented to mitigate fatigue in long COVID patients. Personalized training, including endurance, strength, aerobic, and respiratory exercises, was proven to decrease the level of fatigue [1,4,12,13,14]. Moreover, diet supplementation [15,16], the inhalation of essential oils [17], and high-definition transcranial direct current stimulation [18] were also reported to have a positive effect.

The studies available have mainly focused on exercise capacity in people with long COVID regarding their cardiac and pulmonary properties [19,20]. Meanwhile, in the post-acute phase of SARS-CoV-2 infection, reduced physical performance in COVID-19 convalescents has been reported. Physical performance is a term covering objective measurements of the body’s function related to locomotion, comprising muscle and nervous system function [21]. The Short Physical Performance Battery (SPPB) has been used to assess their potential limitations and classify post-COVID patients according to their physical performance [22,23,24,25,26]. Meanwhile, the Post-COVID-19 Functional Status (PCFS) scale has only been applied to assessing functional status in post-COVID-19 patients [27,28,29]. Functional capacity is defined as an individual’s capability, under controlled conditions, to perform tasks and activities that are necessary or desirable in their lives [30]. No previous study has applied the PCFS scale to evaluating treatment effects in long COVID patients. As a health resort treatment is a complex approach that includes exercises, balneological factors, physical medicine modalities, and climate therapy, it may be recommended for people with persistent COVID-19 symptoms. To date, few theoretical reports have been devoted to health resort treatment of long COVID [31,32,33]. However, Gvozdjáková et al. [34] and Onik et al. [35] assessed the effectiveness of health resort treatment in long COVID patients. This first research concentrated on their clinical symptoms, lung function, and the regeneration of reduced CI-linked platelet mitochondrial respiration. Meanwhile, a second paper retrospectively assessed cardio-pulmonary symptoms in long COVID patients undergoing health resort treatment. Both studies proved that health resort treatment benefits patients facing persistent COVID-19 symptoms.

To our knowledge, no available report has assessed the functional outcomes in long COVID patients using the SPPB and PCFS. This is why our study is unique and contributes to the knowledge in this sense. Moreover, among different types of interventions, health resort treatment is less popular, and no studies have assessed its effectiveness in long COVID patients regarding functional status. This retrospective study had two aims: firstly, to assess the functional status and fatigue level of people with long COVID that qualified for health resort treatment, and, secondly, to assess the effects of health resort treatment in this group of patients.

## 2. Materials and Methods

### 2.1. Research Methods and Data Collection

For this retrospective research, data on patients undergoing health resort treatment because of long COVID at the Rehabilitation Hospital and Sanatorium “Gwarek” in Goczałkowice-Zdrój (Poland) in 2021 were reviewed between March and May 2024. The medical records of 239 patients included demographic data, medical history, a description of the individually tailored treatment course, and scores in the Post-COVID-19 Functional Status (PCFS) scale and the Short Physical Performance Battery (SPPB), as well as their fatigue level before and after the health resort treatment. The Bioethical Committee of the Medical University of Silesia in Katowice stated that this project did not require its opinion (decision number: BNW/NWN/0052/KB/238/23).

### 2.2. Inclusion Criteria

The eligibility criteria for inclusion were the complete medical records of patients who underwent the health resort treatment for long COVID (*n* = 150).

### 2.3. Exclusion Criteria

The following exclusion criteria were established: age above 80 years, neuropsychiatric disorders (multiple sclerosis, post-stroke syndrome, Parkinson’s disease, epilepsy, depression), cardiovascular system diseases (coronary artery disease, heart failure, myocardial infarction in history, percutaneous coronary interventions and/or coronary artery bypass grafting in history, endarterectomy in history, pacemaker, atrioventricular and/or bundle of His blocks, atrial fibrillation, peripheral artery disease), respiratory system disorders (chronic obstructive pulmonary disease, emphysema, pneumoconiosis, asthma), rheumatic diseases (rheumatoid arthritis, ankylosing spondylitis), cancer, lower limb amputations, blindness, Lyme disease, inability to attend general development exercise, and a SPPB score of 0 points at admission.

### 2.4. Treatment Protocol

After the initiation of health resort treatment, all patients attended a complex health resort treatment individually tailored to them, based on a pre-treatment clinical examination. Various treatment methods were used for patients, including balneotherapy, general developmental and respiratory exercises, physical medicine modalities, and health education. All patients performed respiratory exercises. Those exercises aimed to relax the chest muscles, activate lower ribs, prolong exhalation, and aid diaphragmatic breathing. Moreover, each patient performed general development exercises to improve dynamics, endurance, balance, coordination, and muscle strength. The exercises were supervised by a physiotherapist. Routinely, after seven days of treatment, each patient was evaluated to confirm a positive response to the intervention and to exclude paradoxical reactions. Ultimately, patients were discharged after completing the assumed treatment program and final examination.

### 2.5. Methods of Evaluation

Persistent symptoms of COVID-19 and the functional status of people with long COVID were assessed twice: during admission and discharge. The principle of the Polish Post-COVID-19 rehabilitation program recommended the Post-COVID-19 Functional Status (PCFS) as an assessment tool. However, the Short Physical Performance Battery (SPPB) was routinely applied in the sanatorium among the long COVID patients, as most of them were above 60 years of age. The PCFS measures the limitations of daily life caused by COVID-19, considering the last seven days. Patients assessed the severity of the limitation from zero to four points. Zero meant no functional limitations, while four meant an inability to perform daily activities without assistance, i.e., severe limitations. The PCFS has been shown to correlate with the modified Medical Research Council (mMRC) scale [36,37,38,39]. The Short Physical Performance Battery (SPPB) was used to evaluate the physical performance. It consists of the following tasks: a four-minute walking test, a five-repetition sit-to-stand test, and a set of standing balance exercises. Each task can be scored from zero to four points; thus, participants can gain twelve points. This tool is repeatable and sensitive to changes in functionality over time. A score of 0 to 3 points indicates severe physical function disability, 4 to 6 indicates poor function, 7 to 9 indicates moderate function, and 10 to 12 indicates normal function [22,40,41]. Patients also assessed their fatigue on a 0–10 scale, where zero indicated “no symptoms”, and ten meant “the most severe symptoms imaginable”. We have adopted the following method of interpretation: 0–3 points for mild symptoms, 4–7 points for moderate symptoms, and 8–10 points for severe symptoms.

### 2.6. Statistical Analysis

Statistical analysis was performed with STATISICA 13 PL software. Qualitative variables are presented as percentages. Quantitative variables are presented as means with standard deviations. The effect size was calculated using G Power 3.1.94 software. With the assumption of the sample size being 116, α being 0.05 and the statistical power (1-β error probability) being 0.95, the effect size was established as ƒ = 0.315. The data’s normalcy distribution was checked using the Shapiro–Wilk test. As the sample data distribution differed from the normal one, non-parametric tests were applied. Intragroup comparisons were performed using the Wilcoxon signed-rank test. Intergroup comparisons were carried out using the Mann–Whitney U test. The homogeneity of variance was checked with Leven’s test. The Kruskal–Wallis one-way analysis of variance by ranks with a post hoc test was used to perform intergroup comparisons. The level of statistical significance was set to *p* < 0.05. We calculated each variable’s delta (difference between baseline and final measurement) to establish the magnitude of changes.

## 3. Results

The medical records of 116 people (66 women and 50 men) aged 42–79 years (mean age: 64.32 years ± 8.71 years) were analyzed. The patients’ mean body weight was 85.25 kg ± 14.75 kg, their mean body height was 1.67 m ± 0.09 m, and their mean body mass index was 30.42 kg/m^2^ ± 4.76 kg/m^2^. The mean systolic blood pressure of the reviewed patients was 139.86 mmHg ± 13.2 mmHg, and the diastolic blood pressure was 80.2 mmHg ± 7.6 mmHg, measured on admission day. The health resort treatment’s mean duration was 24.46 days ± 6.4 days. The patients’ characteristics are presented in Table 1.

Hypertension was the most common comorbidity in people who qualified for the health resort treatment (58.62% of patients; *n* = 68). In hypertensive patients, the mean systolic blood pressure was 141.66 mmHg ± 13.29 mmHg, while the mean diastolic one was 80.23 mmHg ± 8.25 mmHg at admission. Type 2 diabetes mellitus was noted in 18.97% of patients (*n* = 22), while 12.93% of patients (*n* = 15) were diagnosed with hypothyroidism. Gout coexisted in 3.45% of participants (*n* = 4), and 21.55% of patients (*n* = 25) were diagnosed degenerative joint disease. Only one man (0.9% of the study group) had benign prostatic hyperplasia.

The most frequently applied modality was pneumatic massage (performed in 64.66% of the patients; *n* = 75). Classical massage was prescribed in 6.03% of the patients (*n* = 7). Only one person had lymphatic drainage performed (0.86% of the participants). Among the balneological procedures, whirlpool baths were the most often ordinated (38.79% of the patients; *n* = 45). Pearl baths were applied in 23.28% of the participants (*n* = 27), while mud therapy was applied in 12.93% (*n* = 15). Physical medicine modalities were also prescribed for patients with long COVID. Local cryotherapy was applied in 50% of the subjects (*n* = 58). Light therapy using the infrared wavelength was administered in 41.38% of patients (*n* = 48). Low-level laser therapy was the most often applied physical medicine modality in the study group (60.35% of the patients; *n* = 70). Less frequently, electrotherapy (33.62% of the participants; *n* = 39) and electromagnetic fields (38.79% of the individuals; *n* = 45) were administered in the study group. Only six people attended ultrasounds (5.17% of the patients) during the health resort treatment. All participants received health education on bad habits cessation, healthy diet, physical activity recommendations, and learning the correct technique of using inhalers if requested.

**Figure 1:**
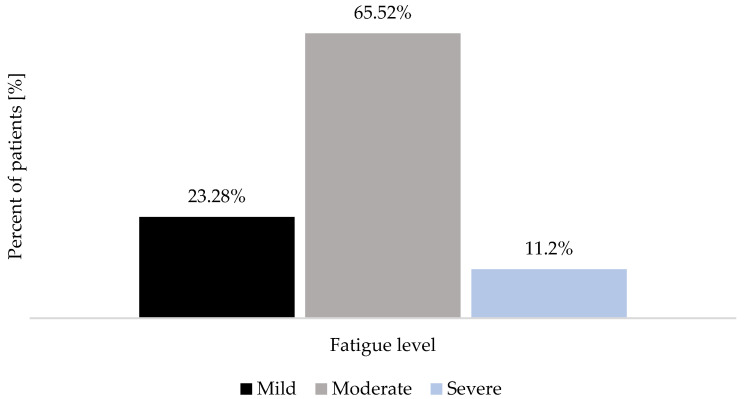
Fatigue severity in patients who qualified for the health resort treatment because of long COVID during pre-treatment measurement.

**Figure 2:**
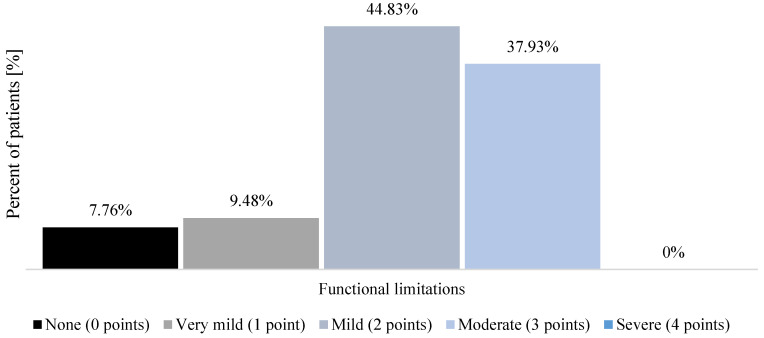
Functional status impairment assessed with PCFS in patients who qualified for the health resort treatment because of long COVID during the pre-treatment measurement.

**Figure 3:**
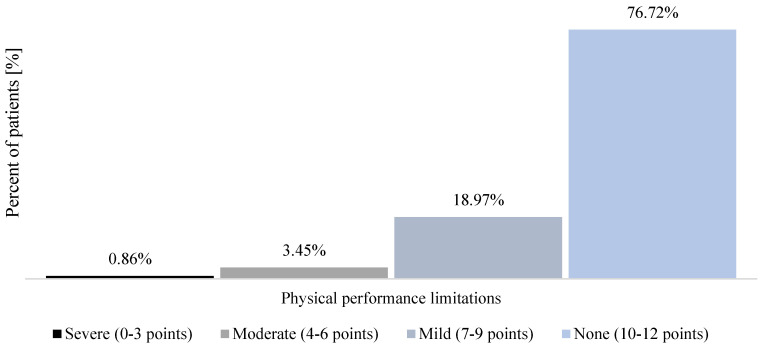
Physical performance limitations assessed with SPPB in patients who qualified for the health resort treatment because of long COVID during the pre-treatment measurement.

When measured before the start of the health resort treatment, most patients declared moderate fatigue (65.52% of the study group; *n* = 76) (Figure 1). The baseline measurement of functional limitations resulting from persistent COVID-19 symptoms performed with PCFS showed that most patients declared mild functional impairment (44.83% of the study group; *n* = 52) (Figure 2). Moreover, most patients (76.72% of the study group; *n* = 89) showed no physical performance limitations, as measured with the SPPB during the pre-treatment assessment (Figure 3).

Patients had a 10% lower mean SPPB score at admission than their post-treatment assessment. At baseline, men scored 8% higher on the SPPB than women. After staying in the sanatorium, the mean SPPB score increased by approximately 12% in women and 8% in men. Moreover, after the health resort treatment, the mean SPPB score was approximately 4% higher in men than women. The average PCFS scores of all patients measured before treatment were 42% higher than after their stay in the sanatorium. Moreover, the PCFS score in women was 13% higher than in men. During the second measurement, the mean PFCS score decreased by approximately 45% in women and about 37% in men. At admission, the level of fatigue was 13% higher in women than in men. Both women and men declared a significant reduction in the severity of this symptom as a result of health resort treatment. Fatigue was reduced by approximately 56% in women and about 63% in men. In the study group, the health resort treatment reduced fatigue by about 55% (Table 2). The calculation of the magnitude of change revealed that women had greater treatment-related changes in measured variables, although these were not statistically significant. The highest gender difference was noted in ΔSPPB; it was 29% higher in women than in men. The lowest difference between women and men was noted in the Δfatigue (15% difference) (Table 3). 

In the next statistical analysis stage, the study group was divided into three subgroups depending on age. Group I constituted people aged 40–59 years (*n* = 35). Group II comprised people aged 60–69 (*n* = 40). Patients with an age range of 70–79 were recruited into group III. During the pre-treatment assessment, people aged 40–59 had about 10% better SPPB scores than patients aged 70–79. Nevertheless, the health resort treatment used in each age group significantly improved the average SPPB score, with the highest rise observed in group III (14%). Mean PCFS scores decreased by about 42% in each group. The decrease in fatigue was comparable in all age groups. In group I, fatigue was reduced by about 54%, in group II by 56%, and in group III by 57% (Table 4). The analysis of the effectiveness of the health resort treatment revealed that, in people aged 40–59 years (group I), Δ SPPB was 45% higher than in patients aged 70–79 years. The remaining delta variables did not differ significantly between groups of patients undergoing health resort treatment (Table 5).

## 4. Discussion

In people who qualified for the health resort treatment because of long COVID, the majority declared a moderate level of fatigue (65.52% of the study group) at admission. Fatigue assessment was performed with a numeric rating scale (0–10 points), commonly used for self-reporting symptoms [42]. Available reports indicate different incidences of fatigue in patients with persistent COVID-19 symptoms [1,2,3,4]. In our study, 88% of participants reported fatigue (≥1 point) during the pre-treatment assessment. Our results are inconsistent with data provided by Nopp et al. [1], Koc et al. [2], Chuang et al. [4], Ceban et al. [43], and Besnier et al. [44], who reported lower incidences. Oldenmenger et al. [45] suggest different cut points for fatigue. With the assumption of the cut-off point being ≥ four, clinically relevant fatigue was noted in 76.62% of patients enrolled in the study. This is in accordance with Lippi et al. [5], who reported a 78% incidence of fatigue in COVID-19 convalescents. Ceban et al. [43] and Bai et al. [46] indicate more significant fatigue in women with long COVID. During the baseline measurement, the level of fatigue did not differ significantly between genders, although it was 13% higher in women. Women are twice likely to develop long COVID because of their elevated immune response. This may explain the differences noted between the genders [47]. Even though we did not perform biochemical analyses that would allow us to assess the functioning of the immune system, this might be a direction for further studies to take.

Available studies have evidenced a reduced physical performance in COVID-19 survivors [25]. Moreover, Rahimi et al. [48] reported a reduced level of physical performance in COVID-19 convalescents, compared to healthy people without a history of SARS-CoV-2 infection. In the study group, 76.72% of patients had no physical performance limitations, assessed at admission using the SPPB. This is in accordance with Baricich et al. [26], who report that 86% of COVID-19 survivors obtained a SPPB score of > 10, measured about 125 days after discharge. People with a history of COVID-19 are prone to experiencing balance disorders [49,50], might be at a greater risk of falls, experience gait disturbances [51], and face muscle weakness [52]. Since SPPB evaluates physical performance in three domains, namely balance, strength, and gait [53], it addresses the conditions of long COVID patients. The SPPB score was about 8% higher during pre-treatment measurement in men than in women (*p* < 0.01). Our data are comparable to those presented by Ramírez-Vélez et al. [54], who reported that the total SPPB score was 0.85 points higher in men than women.

The utility and value of the PCFS scale have been previously confirmed [37,38]. In the study group, before initiating the health resort treatment, most patients (44.83%) declared mild functional limitations, while 37.39% of them reported moderate ones. Our results are opposite to those of Pant et al. [28], who stated that, in COVID-19 survivors, 56.6% reported no functional limitations. Moreover, Taboada et al. [55] also found that most COVID-19 convalescents did not complain of functional limitations six months after the SARS-CoV-2 infection clearance. In women who qualified for the health resort treatment, we found that their PCFS scores were 13% higher than those of men. Until now, no available report has compared the functional status of long COVID women and men. Bai et al. [46] and Sylvester et al. [56] postulate that long COVID occurs more often in women as they are more likely to develop neurological and musculoskeletal disorders, which may explain the results obtained in the study group.

The health resort treatment improved the functional status (decrease in PCFS scale by about 41%) and physical performance (increase in SPPB by approximately 10%) in the study group. However, the level of fatigue decreased after the curation (about 55%). During their stay in the sanatorium, patients were exposed to balneological factors, including different baths with mineral water and mud therapy. The effects of hydrotherapy depend on the temperature; cold water reduces pain and stimulates peripheral vasoconstriction and subsequent vasodilatation, while hot water causes vasodilatation and decreases muscle tone [57]. Moreover, mineral waters and muds have anti-inflammatory and antioxidant effects [58]. The anti-inflammatory effect results from reducing pro-inflammatory cytokines and increasing anti-inflammatory ones [59]. In addition to balneological factors, people attending the health resort treatment also had a massage. In the study group, pneumatic massage was performed on 64.66% of patients. This modality promotes blood vessel dilation, improves flexibility, reduces pain, and stimulates regeneration [60,61,62]. Wheibe et al. [63] suggest that massage therapy may effectively reduce inflammatory markers. One of the long COVID hypotheses assumes that chronic inflammation and immune system dysregulation are key reasons [4]; this is why balneological factors and massage may be adjuvant. The healing properties of climate also support the action of balneological factors [64,65]. Kanayama et al. [66] inform that endurance walking exercises are a solid part of climate therapy and also play a role in positive responses to the health resort treatment in long COVID patients. While staying in a health resort, patients could walk in their free time in green areas, which may have also had a positive impact.

It is postulated that SARS-CoV-2 infection may lead to the inflammation of myofibers and neuromuscular junctions with secondary fatigue and diminishment of exercise tolerance [4]. In our study, all participants attended respiratory and general development exercises. They have been proven to act beneficially in post-COVID patients, decreasing fatigue and improving exercise capacity [1,12]. This is in accordance with our results, as we observed a reduced fatigue level after the health resort treatment. Scurati et al. [67] postulate that skeletal muscle atrophy and diminished physical performance may be observed in long COVID patients, while Ghiotto et al. [68] suggest that exercises may improve physical performance. In the study group, the SPPB score improved after curation in the sanatorium, as all patients attended supervised exercises. Treatment in the sanatorium significantly improved patients’ functional status, as assessed with the PCFS scale. Tsekoura et al. [69] claim that, in patients recovering from COVID-19, multidisciplinary interventions are essential for functional improvement. Treatment in the health resort comprises balneological factors, climate action, physical medicine modalities, exercises, and health education. Various factors make the health resort treatment complex; thus, it may be effectively applied in long COVID patients [31,32,33] and explain the observed improvement in functional status. A reduction in fatigue may also be a consequence of this complex action.

As women are more likely to develop long COVID [46,47], we have also considered the effects of the health resort treatment with dependency on gender. During the post-treatment measurement, the only difference between women and men was their SPPB scores, which were about 4% lower in women than in men. Nevertheless, considering ΔSPPB, ΔPCFS, and Δfatigue, health resort treatment impacts women and men comparably, as they did not differ significantly. Age is also associated with long COVID symptoms severity in convalescents [4,5,6], which is why we decided to divide the study group into age subgroups. During the pre-treatment measurement, the SPPB score was about 4% higher in patients aged 40–59 years than in those aged 70–79 (*p* < 0.05). The noted differences might arise from aging, which is associated with diminished balance, muscular strength, and walking speed [70]. It might also be a consequence of SARS-CoV-2 infection. However, we did not measure the participants’ physical performance after infection clearance. After the curation in a sanatorium, no significant differences were found between age groups. The assessment of the treatment’s effectiveness, expressed as delta variables, revealed that ΔSPPB in the oldest participants was lower than in those aged 40–59. It indicates that people aged 70–79 responded better to treatment and had a clear improvement in physical performance compared to the youngest participants. Until now, it has been proved that health resort treatment acts effectively in the geriatric population [71,72,73]. This supports our results and confirms the usefulness of the health resort treatment in this group of long COVID patients. Moreover, the analysis of Δfatigue revealed no differences between the age groups, even though fatigue levels decreased in each of them after curation. This suggests that health resort treatment mitigates fatigue regardless of age.

In people who qualified for the health resort treatment, the most frequent comorbidity was hypertension (58.62% of participants). Type 2 diabetes mellitus was noted in 18.97% of patients. Previous studies devoted to rehabilitative interventions in long COVID also included people with hypertension and type 2 diabetes mellitus [1,12,28]. This supports our study group’s composition. Moreover, hydrotherapy and health resort treatment have been proven beneficial in patients with those comorbidities [57,74,75]. Although we did not assess the blood pressure after the health resort treatment, positive vascular reaction to applied treatment might also have impacted improvement in long COVID patients. In total, 21.55% of participants were diagnosed with degenerative joint disease. Patients had administered physical medicine modalities mainly because of joint pain. Those modalities have been proven to relieve pain, have an anti-inflammatory effect, reduce edema, improve local blood flow, improve regeneration, and stimulate tissue proliferation [35]. The numerous biological effects of physical factors applied in the treatment may also positively affect patients’ condition and improve the outcomes.

## 5. Study Limitations and Further Studies Directions

Our study has some limitations. Firstly, it was impossible to determine the direct time from the end of SARS-CoV-2 infection to the start of spa treatment because Polish legal regulations did not precisely define this time interval. It was only defined that convalescents should begin the curation within twelve months of recovery from COVID-19. Secondly, fatigue assessment was assessed using a numeric rating scale. In future studies, using other tools, such as the Fatigue Rating Scale, to obtain solid data would be prudent. Thirdly, depending on the clinical condition, each patient had a personalized treatment course. In future studies, treatment courses should be designed prior to the recruitment of the patients. It would enable the effectiveness of different treatment strategies to be assessed for long COVID. Finally, we recommend applying the 6-minute walk test to complement PCFS and SPPB in future studies.

## 6. Conclusions

Fatigue was of moderate intensity in the long COVID patients that qualified for the health resort treatment. Most of the long COVID patients reported mild functional limitations, whereas their physical performance was undisturbed. Health resort treatment improved functioning in patients with persistent COVID-19 symptoms by reducing fatigue and improving their functional capacity and physical performance. It should be recommended as a supplement to the standard treatment because of its complexity.

## Figures and Tables

**Table 1:** Characteristics of patients undergoing the health resort treatment for long COVID.

	Women (*n* = 66)			Men (*n* = 50)		
	Min	Max	Mean ± SD	Min	Max	Mean ± SD
Age [years]	43	77	64.91 ± 8.63	42	79	63.54 ± 8.84
Body weight [kg]	55	120	81.32 ± 15.27	70	120	90.44 ± 12.38
Body height [m]	1.5	1.78	1.62 ± 0.06	1.61	1.95	1.75 ± 0.07
BMI [kg/m^2^]	21.45	44.08	30.97 ± 5.46	23.99	39.79	29.7 ± 3.55
Systolic blood pressure [mmHg]	105	160	139.41 ± 14.25	115	165	140.44 ± 11.8
Diastolic blood pressure [mmHg]	55	95	79.2 ± 8.22	66	95	81.52 ± 6.54
Treatment duration [day]	19	47	24.97 ± 6.83	17	46	23.78 ± 5.78

**Table 2:** The efficacy of the health resort treatment in people with long COVID.

**Short Physical Performance Battery (SPPB) [Points]**	**Pre-Treatment Measurement**			**Post-Treatment Measurement**			***p* Value ^1^**
	**Min**	**Max**	**Mean ± SD**	**Min**	**Max**	**Mean ± SD**	
Whole group (*n* = 116)	3	12	10.41 ± 1.84	6	12	11.57 ± 0.94	*p* < 0.00001
Women (*n* = 66)	3	12	10.02 ± 1.89	6	12	11.35 ± 1.14	*p* < 0.00001
Men (*n* = 50)	4	12	10.92 ± 1.65	10	12	11.86 ± 0.45	*p* < 0.00001
*p* value ^2^	*p* < 0.01			*p* < 0.01			
**Post-COVID-19 Functional Status (PCFS) [points]**	**Pre-Treatment Measurement**			**Post-Treatment Measurement**			***p* Value ^1^**
	**Min**	**Max**	**Mean ± SD**	**Min**	**Max**	**Mean ± SD**	
Whole group (*n* = 116)	0	3	2.13 ± 0.88	0	2	1.23 ± 0.62	*p* < 0.00001
Women (*n* = 66)	0	3	2.26 ± 0.73	0	2	1.26 ± 0.54	*p* < 0.00001
Men (*n* = 50)	0	3	1.96 ± 1.03	0	2	1.2 ± 0.73	*p* < 0.00001
*p* value ^2^	*p* > 0.05			*p* > 0.05			
**Fatigue [points]**	**Pre-Treatment Measurement**			**Post-Treatment Measurement**			***p* Value ^1^**
	**Min**	**Max**	**Mean ± SD**	**Min**	**Max**	**Mean ± SD**	
Whole group (*n* = 116)	0	10	4.83 ± 2.38	0	6	2.15 ± 1.31	*p* < 0.00001
Women (*n* = 66)	0	9	5.12 ± 2.13	0	6	2.24 ± 1.23	*p* < 0.00001
Men (*n* = 50)	0	10	4.46 ± 2.63	0	6	2.02 ± 1.42	*p* < 0.00001
*p* value ^2^	*p* > 0.05			*p* > 0.05			

Legend: *p* value ^1^—intragroup comparison; *p* value ^2^—intergroup comparison (women vs. men).

**Table 3:** Comparison of the magnitude of changes mediated by the health resort treatment in women and men with long COVID.

	**Δ Short Physical Performance Battery (SPPB) [Points]**		
	**Min**	**Max**	**Mean ± SD**
Women (*n* = 66)	−4	1	−1.33 ± 1.42
Men (*n* = 50)	−8	0	−0.94 ± 1.48
*p* value	*p* > 0.05		
	**Δ Post-COVID-19 Functional Status (PCFS) [points]**		
	**Min**	**Max**	**Mean ± SD**
Women (*n* = 66)	0	2	1.00 ± 0.74
Men (*n* = 50)	0	2	0.76 ± 0.75
*p* value	*p* > 0.05		
	**Δ fatigue [points]**		
	**Min**	**Max**	**Mean ± SD**
Women (*n* = 66)	0	6	2.88 ± 1.5
Men (*n* = 50)	0	6	2.44 ± 1.7
*p* value	*p* > 0.05		

Legend: **Δ**—magnitude of changes, *p* value—intergroup comparison (women vs. men).

**Table 4:** The efficacy of the health resort treatment in age-dependent groups of patients with long COVID.

**Short Physical Performance Battery (SPPB) [Points]**	**Pre-Treatment Measurement**			**Post-Treatment Measurement**			***p* Value ^1^**
	**Min**	**Max**	**Mean ± SD**	**Min**	**Max**	**Mean ± SD**	
Group I (*n* = 35)	4	12	10.94 ± 1.81	10	12	11.80 ± 0.53	*p* < 0.01
Group II (*n* = 40)	7	12	10.53 ± 1.54	8	12	11.55 ± 0.96	*p* < 0.0001
Group III (*n* = 41)	3	12	9.83 ± 2.01	6	12	11.39 ± 1.14	*p* < 0.0001
*p* value ^2^	*p* < 0.01			*p* > 0.05			
Post hoc test	Group I > Group III (*p* = 0.005)						
**Post-COVID-19 Functional Status (PCFS) [points]**	**Pre-Treatment Measurement**			**Post-Treatment Measurement**			***p* Value ^1^**
	**Min**	**Max**	**Mean ± SD**	**Min**	**Max**	**Mean ± SD**	
Group I (*n* = 35)	0	3	2.17 ± 0.89	0	2	1.26 ± 0.66	*p* < 0.00001
Group II (*n* = 40)	0	3	2.15 ± 0.95	0	2	1.25 ± 0.63	*p* < 0.00001
Group III (*n* = 41)	0	3	2.07 ± 0.82	0	2	1.2 ± 0.6	*p* < 0.00001
*p* value ^2^	*p* > 0.05			*p* > 0.05			
**Fatigue [points]**	**Pre-Treatment Measurement**			**Post-Treatment Measurement**			***p* Value ^1^**
	**Min**	**Max**	**Mean ± SD**	**Min**	**Max**	**Mean ± SD**	
Group I (*n* = 35)	0	10	5.43 ± 2.38	0	6	2.49 ± 1.38	*p* < 0.00001
Group II (*n* = 40)	0	9	4.8 ± 2.26	0	4	2.13 ± 1.18	*p* < 0.00001
Group III (*n* = 41)	0	8	4.37 ± 2.4	0	6	1.88 ± 1.35	*p* < 0.00001
*p* value ^2^	*p* > 0.05			*p* > 0.05			

Legend: *p* value ^1^—intragroup comparison; *p* value ^2^—intergroup comparison.

**Table 5:** Comparison of the magnitude of changes mediated by the health resort treatment in age-dependent groups of patients with long COVID.

	**Δ Short Physical Performance Battery (SPPB) [Points]**			***p* Value**	**Post Hoc Test**
	**Min**	**Max**	**Mean ± SD**		
Group I (*n* = 35)	−8	1	−0.86 ± 1.63	*p* < 0.05	Group I > Group III(*p* = 0.03)
Group II (*n* = 40)	−4	2	−1.03 ± 1.12		
Group III (*n* = 41)	−4	1	−1.56 ± 1.52		
	**Δ Post-COVID-19 Functional Status (PCFS) [points]**			***p* value**	**Post Hoc Test**
	**Min**	**Max**	**Mean ± SD**		
Group I (*n* = 35)	0	2	0.91 ± 0.78	*p* > 0.05	-
Group II (*n* = 40)	0	2	0.9 ± 0.71		
Group III (*n* = 41)	0	2	0.88 ± 0.78		
	**Δ fatigue [points]**			***p* value**	**Post Hoc Test**
	**Min**	**Max**	**Mean ± SD**		
Group I (*n* = 35)	0	6	2.94 ± 1.57	*p* > 0.05	-
Group II (*n* = 40)	0	6	2.67 ± 1.62		
Group III (*n* = 41)	0	6	2.49 ± 1.61		

Legend: **Δ**—magnitude of changes; *p* value—intergroup comparison (between age groups).

## Data Availability

The data that support the findings of this study are available from the corresponding author.
